# Whether and When Could Generative AI Improve College Student Learning Engagement?

**DOI:** 10.3390/bs15081011

**Published:** 2025-07-25

**Authors:** Fei Guo, Lanwen Zhang, Tianle Shi, Hamish Coates

**Affiliations:** 1 School of Education, Tsinghua University, Haidian District, Beijing 100084, China; feiguo0121@tsinghua.edu.cn (F.G.); stl22@mails.tsinghua.edu.cn (T.S.); 2 Policy Research and Planning Office, Tsinghua University, Haidian District, Beijing 100084, China; zhanglw22@mails.tsinghua.edu.cn; 3 Crawford School of Public Policy, Australian National University, Canberra, ACT 2601, Australia

**Keywords:** generative AI, effective teaching practices, college student learning engagement

## Abstract

Generative AI (GenAI) technologies have been widely adopted by college students since the launch of ChatGPT in late 2022. While the debate about GenAI’s role in higher education continues, there is a lack of empirical evidence regarding whether and when these technologies can improve the learning experience for college students. This study utilizes data from a survey of 72,615 undergraduate students across 25 universities and colleges in China to explore the relationships between GenAI use and student learning engagement in different learning environments. The findings reveal that over sixty percent of Chinese college students use GenAI technologies in Academic Year 2023–2024, with academic use exceeding daily use. GenAI use in academic tasks is related to more cognitive and emotional engagement, though it may also reduce active learning activities and learning motivation. Furthermore, this study highlights that the role of GenAI varies across learning environments. The positive associations of GenAI and student engagement are most prominent for students in “high-challenge and high-support” learning contexts, while GenAI use is mostly negatively associated with student engagement in “low-challenge, high-support” courses. These findings suggest that while GenAI plays a valuable role in the learning process for college students, its effectiveness is fundamentally conditioned by the instructional design of human teachers.

## 1. Introduction

In recent decades higher education has experienced a period of unprecedented global expansion. Gross enrollment ratios have risen significantly across most regions, marking the transition of higher education systems from elite to mass and, in some cases, to universal access (Trow, 2007). While this expansion has broadened educational opportunities, it has also caused increasing concerns about educational quality and student learning outcomes (Altbach et al., 2019). One of the major challenges lies in the growing heterogeneity of the student population, which has led to a widening gap in students’ academic preparedness before entering university (Al-Maskari et al., 2022). Many students struggle to adapt to university-level curricula that assume a high level of prior knowledge and academic competence (Karp & Bork, 2014). Simultaneously, large class sizes—often a byproduct of rapid enrollment growth—have made it difficult for instructors to provide personalized guidance and support, thereby hindering the development of higher-order thinking, mastery of complex knowledge, and student skill development (Carrell & West, 2010; Taft et al., 2019).

Against this backdrop, the emergence and diffusion of generative artificial intelligence (GenAI) technologies such as ChatGPT and Deepseek have sparked new optimism about enhancing learning in massified higher education. Compared to traditional instructional models, GenAI offers transformative educational possibilities by providing scalable, on-demand, and personalized support. These tools deliver immediate feedback, adaptive explanations, and tailored learning pathways to meet diverse student needs, while also assisting with problem-solving tasks and automating routine academic processes (S. Guo et al., 2024; Smolansky et al., 2023). Such capabilities are particularly valuable in large-enrollment or under-resourced contexts where individualized instructional support is limited.

However, this technological promise is accompanied by mounting concerns. The ease of access to GenAI tools may inadvertently undermine students’ self-regulation and intrinsic motivation for learning. Excessive reliance on GenAI for completing assignments or generating content can undermine students’ writing skills and creativity, reduce their cognitive engagement, and lead to shallow learning (Cotton et al., 2023). Moreover, the boundary between appropriate academic assistance and cheating becomes increasingly blurred, posing ethical and pedagogical challenges for both students and educators (Susnjak & McIntosh, 2024).

Despite growing interest in the educational applications of GenAI, empirical evidence on its actual use and impact in higher education remains limited and fragmented. Existing quantitative research often relies on small, non-representative samples and is mostly descriptive. For example, Baek et al. (2024) surveyed 1001 U.S. college students on their perceptions and use of ChatGPT, while Habibi et al. (2023) examined influencing factors among 1172 Indonesian students. Bassner et al. (2024) is among the few studies exploring the usefulness of AI-powered tools, which used interaction data from 221 students with Iris and questionnaire data from 121 of these students to find that students can use AI-powered tools to clarify concepts outside the classroom, simulate problem-solving processes, or draft and refine academic writing, all in a one-on-one, interactive format. Overall, the specific effects of GenAI on different dimensions of student learning remain under-explored, and studies based on large, representative samples are particularly scarce.

Another less investigated perspective of GenAI used in higher education is the context of when GenAI facilitates student learning. Prior studies have shown that the impact of educational technologies is not uniform but depends on how they are integrated into instructional environments (Keuning et al., 2024; Zhao et al., 2002). Therefore, it is necessary to consider the context of use when examining the impact of GenAI. Yet there is little evidence in this regard shown in previous studies.

This study is situated in the context of China, where the relevance and urgency of these questions are particularly salient. China’s higher education system has just entered the universal stage of participation, with the world’s largest student population (MOE, 2023). Many institutions face increasing difficulties in maintaining instructional quality amid scale expansion, especially in terms of effective student–faculty interaction and curriculum adaptation (Zhou, 2019). Meanwhile, China has emerged as a global leader in AI development, particularly in terms of the number of publications and patents in AI-related fields (Min et al., 2023). Surveys also suggest that Chinese students are open and enthusiastic about the integration of AI into learning (Tian et al., 2024). Consistent with these findings, the survey data used in this study show that 64.47% of participants reported using GenAI technologies in Academic Year 2023–2024, indicating a relatively high adoption rate among university students in China. These unique characteristics make China a particularly informative setting to examine how GenAI shapes student learning experiences in a mass higher education system.

Drawing upon a large-scale national survey on college student learning and development in China, this study focuses on the role of GenAI in college student learning. In particular, we examine whether and in what context GenAI facilitates student engagement in college. Student engagement is a multidimensional construct encompassing behavioral, emotional, and cognitive dimensions of involvement in learning (Coates, 2005; Fredricks et al., 2004; Kahu, 2013). As a construct grounded in the active investment of time, effort, and psychological resources, engagement reflects students’ self-regulated and agentic involvement in learning processes (Fredricks et al., 2004), thereby serving as a key proxy for students’ earning initiative and a critical indicator of educational quality and student success (Gao et al., 2022; Kuh et al., 2006). We pay special attention to student engagement in an academic context as characterized by the level of academic challenges and effectiveness of faculty support in courses. This is because student engagement can be stimulated by the effective design and delivery of educational activities, the core of which lies in the combination of challenge and support (Coates, 2005; Kuh et al., 2011; Trowler, 2010; Shi et al., 2024).

Specifically, this paper asks three research questions (RQ):

RQ 1. What is the overall situation regarding the use of GenAI among undergraduate students in China?

RQ 2. What is the association between the use of GenAI and students’ learning engagement in college?

RQ 3. Is there any difference among students in different learning contexts as characterized by the level of academic challenge and support from professors?

By answering the above questions, this paper aims to contribute to the debates of whether GenAI facilitates or hinders learning. The first question describes the popularity of GenAI among Chinese undergraduate students and students’ satisfaction with the usefulness of GenAI. The second explores the overall relationships between student learning engagement and the frequency and satisfaction of GenAI. The last question compares such relationships in different learning contexts.

## 2. Theoretical Hypotheses

Although the three RQs are all descriptive in nature, we propose four hypotheses based on previous theories and empirical studies:

**Hypothesis 1.** 
*The more frequent use and higher satisfaction of GenAI, the more active a student participates in learning activities.*

This refers to the behavioral dimension of student engagement, which includes activities such as note-taking in class, contributing to classroom discussion, on-time review and summarizing. Previous studies demonstrate the capability of GenAI in assisting routine tasks such as note-taking, summarizing materials, and translation between different languages (Beveridge et al., 2025; Kwok et al., 2025). If GenAI makes such tasks easier, students may be more willing to become involved in such activities in class.

**Hypothesis 2.** 
*The more frequent use and higher satisfaction of GenAI, the deeper a student engages in higher-order learning.*

This refers to the cognitive dimension of student engagement. Constructivist learning theory emphasizes learners’ active construction of meaning through interactive and customized dialogues (Koschmann, 1999). Through personalized learning materials, meaningful task design, and targeted instructional scaffolding, GenAI can stimulate higher-order cognitive processes (Ansari et al., 2023; Lin et al., 2024) and therefore holds the potential to serve as an effective facilitator of cognitive learning engagement.

**Hypothesis 3.** 
*The more frequent use and higher satisfaction of GenAI, the more positive a student’s attitude towards learning.*

This refers to the emotional dimension of student engagement. From an educational psychology perspective, GenAI’s assistance can mitigate a student’s stress and mental workload associated with academic challenges, potentially enhancing students’ self-efficacy in their learning endeavors (Ling & Junzhou, 2025). This is evident in existing studies finding that AI-powered chatbots and virtual mentors are crucial in offering emotional support, effectively reducing students’ anxiety levels (Li & Jan, 2023).

**Hypothesis 4.** 
*The associations between GenAI and students’ learning are more salient in high-challenging contexts than in low-challenging contexts, but more profound in less-supportive contexts than highly supportive contexts.*

The assumption under this hypothesis is that GenAI, in general, plays the role of a facilitator in learning and is complementary to the support from faculty members.

Hypotheses 1 to 3 are relevant to RQ2, and Hypothesis 4 is relevant to RQ3.

## 3. Method

### 3.1. Data and Sample

This study uses data from the 2024 China College Student Survey, which focused on student engagement in college. The sample consists of undergraduate students from 24 universities and colleges who volunteered to participate in CCSS, including 11 Double World-Class (DWC) higher education institutions (HEIs)^1^ and 14 non-elite ones. Stratified random sampling was used in each institution to draw participants. The valid returned sample contains 72,615 students with an average response rate of 70.76%. Table 1 presents the detailed characteristics of the valid sample.

### 3.2. Variables

The regular CCSS questionnaire contains 10 sections, including students’ learning behaviors in class, attitudes towards learning, perception on effective teaching activities, interactions with faculty, peers and other people on campus, participation in extra-curricular activities, perception on overall institutional environment, perception on learning gains, satisfaction with the institution and college life, pre-college experience, and demographic information. In 2024, we added a module regarding GenAI to learn about students’ experience, feelings, and attitudes towards using GenAI in daily and academic tasks. The variables used in this study were constructed with information collected through the CCSS 2024 questionnaire, which asked about student experience in Academic Year (AY) 2023–2024. The definition, measurement, and descriptive statistics of all variables are presented in Table A1, along with the Cronbach’s alpha for reliability of the constructed scales^2^.

Using students’ ratings of academic challenge and support from faculty members, we construct a categorical variable to describe the learning context. Based on whether the score of the specific variable (level of academic challenge and faculty support) is above or below the mean, students’ learning context is categorized into four types: “low support, low challenge” (N = 28,294, 38.96%), “low support, high challenge” (N = 4901, 6.75%), “high support, low challenge” (N = 8151, 11.22%), “high support, high challenge” (N = 31,269, 43.06%).

### 3.3. Models

To answer RQ1, we first use descriptive analysis to present the frequency of using GenAI and satisfaction with its usefulness among students, and then use ANOVA analysis to examine the differences in the use of GenAI in different learning contexts.

RQ2 and related Hypotheses 2 to 4 ask about the association between the use of GenAI and students’ learning engagement. We use multiple regressions to examine the hypotheses with the following Model 1:

(1) $${SLE}_{i}=\beta_{0}+\beta_{1}{AcdUse}_{i}+\beta_{2}{LifeUse}_{i}{+\beta }_{3}\;{Context}_{i}+\beta_{4}{Covariates}_{i}+\varepsilon$$

where *SLE* refers to student learning engagement, including behavioral engagement (*BE*), cognitive engagement (*CE*), and emotional engagement (*EE*) as defined in Table A1. In addition, as emotional engagement is a complex construct, we also use the second-level dimensions under this construct: self-efficacy in mastering academic tasks (EE_SE), interest in learning (EE_IL), resilience towards difficulties in learning (EE_AR), and intensity of learning motivation (EE_MI). The variables are used as the dependent variable one by one.

Among the independent variables, *Acduse* refers to the frequency of using GenAI in an academic setting and satisfaction with its usefulness. $\beta_{1}$ is the coefficient of interest to answer RQ2. *Lifeuse* refers to the frequency of using GenAI and satisfaction with its usefulness in daily life. This is to control for students’ general perceptions of GenAI. *Context* consists of a series of dummy variables indicating the four categories of learning context. The “low support, low challenge” group is used as the reference group in regressions. *Covariates* refer to students’ demographic variables as listed in Table 1, and social desirability tendency (SD) when filling out the CCSS questionnaire. It is necessary to control for social desirability in regression models to address the bias induced by self-reported data (F. Guo et al., 2018).

RQ 3 and Hypothesis 5 further examine the differences in the associations between GenAI and learning engagement across different learning contexts. Therefore, an interaction term is added to the model:

(2) $${SLE}_{i}=\beta_{0}+\beta_{1}^{\prime }{AcdUsage}_{i}*{Context}_{i}+\beta_{2}{LifeUsage}_{i}{+\beta }_{3}\;{Context}_{i}+\;\beta_{4}{Covariates}_{i}+\varepsilon ,$$

As *Context* is a series of dummy variables, $\beta_{1}^{\prime }$ in Model 2 indicates the association between the use of GenAI in academic settings in each type of learning context.

The above models were estimated with Ordinary Least Square (OLS) regression. Continuous variables are standardized before entering the models. The missing flag method (Jones, 1996) was employed to handle missing data in the independent variables. Standard errors in all the above models were clustered at the higher education institution to adjust for the potential correlation between individuals from the same institution.

## 4. Results

### 4.1. The Prevalence and Satisfaction of College Students Using GenAI

#### 4.1.1. The Frequency of Using GenAI

**Figure 1:**
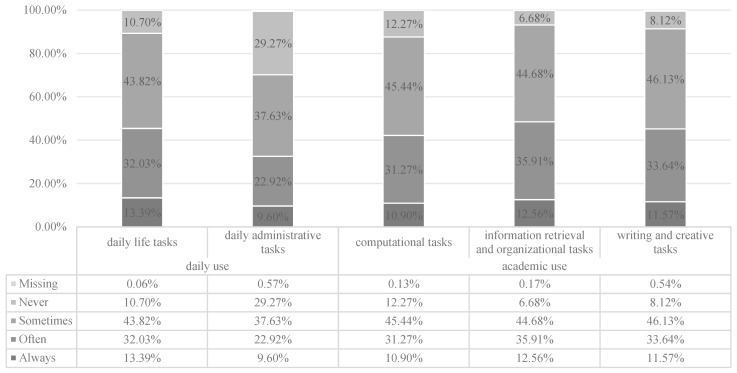
The frequency of college students using GenAI for five types of tasks (N = 46,818).

64.47% of the participants in the CCSS 2024 survey reported using GenAI technologies (N = 46,818). Figure 1 presents a summary of the frequency of using GenAI in daily life (including tasks such as searching for information to meet living demands and handling administrative requirements) and academic settings (including computational tasks, information retrieval and synthesis, and writing or creative tasks). The results suggest that among students who have used GenAI, no more than 50% use it frequently (“Always” and “Often”). Using GenAI in academic research is more common than in daily life. The most frequently used scenarios to use GenAI are information retrieval and organization, and academic writing, while the least used scenario is handling daily administrative work.

#### 4.1.2. Satisfaction with the Usefulness of GenAI

**Figure 2:**
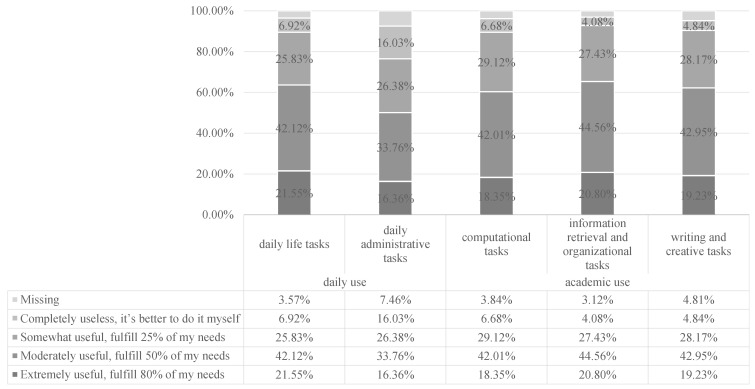
College students’ perceptions of the usefulness of GenAI (N = 46,818).

Figure 2 presents college students’ perceptions of how GenAI is helpful in dealing with the task, implying their satisfaction with the usefulness of GenAI. Overall, about 60% of students who have used GenAI consider it moderately useful in both daily and academic contexts (fulfilling about 50% of their needs). Among the various scenarios, GenAI was rated most useful for information retrieval in both academic and daily life settings. This implies that Chinese students consider GenAI more as a comprehensive search engine than as an intelligent assistant in creative tasks.

#### 4.1.3. Academic Use of GenAI Across Different Learning Contexts

To examine the overall frequency and satisfaction of GenAI use in the academic setting, we constructed two continuous variables (Studyfre and Studyhelp, respectively) by taking the mean scores across the responses to the corresponding three items in academic settings. Table 2 illustrates the comparison of the standardized scores across the four learning contexts.

Overall, students reported a significantly higher frequency of GenAI academic use in high-challenge contexts compared to low-challenge contexts, regardless of the support level. This indicates that students tend to use GenAI as a tool to cope with academic challenges, and this tendency is unrelated to their perceived level of support. Students’ satisfaction with the assistance provided by GenAI in academic settings is also higher in high-challenge contexts than in low-challenge contexts. But there is also a significant gap between the “low support, high challenge” and “high support, high challenge” contexts. This suggests that students’ perception of the usefulness of GenAI is combinedly influenced by the level of challenge and support.

### 4.2. The Impacts of GenAI Use on Student Engagement

Table 3 reports the regression coefficients of Model 1. The key independent variable in Panel 1 is a binary variable indicating whether the students ever used GenAI. Therefore, the coefficients show the gaps in learning engagement between college students who use GenAI and those who do not. Students using GenAI report more cognitive engagement and psychological engagement, especially in self-efficacy and academic resilience, after controlling for the influence of learning contexts, demographic backgrounds and social desirability.

The key independent variables in Panel 2 are the frequency and satisfaction with GenAI in academic and daily life settings. As shown in the table, the use of GenAI in daily life is significantly positively correlated with learning engagement across all dimensions. In learning scenarios, however, the frequency of GenAI use and the satisfaction of its usefulness show mixed positive and negative correlations: in terms of behavioral engagement: both the frequency of use and the perception of usefulness are negatively correlated; in terms of cognitive engagement: the frequency of use is positively correlated, while the perception of usefulness is not significant; and in terms of emotional engagement: the frequency of use is not significant, while the perception of usefulness is positively correlated.

As for the specific sub-dimensions of emotional engagement, when controlling for the use of GenAI in daily life, the frequency of GenAI use in academic contexts shows significant negative relationships with learning interest (*p* < 0.05) and motivational level (*p* < 0.001), but concurrently presents significant positive relationships with academic resilience (*p* < 0.01). The satisfaction of GenAI’s assistance in academic tasks is significantly and positively correlated with self-efficacy (*p* < 0.01) and academic resilience (*p* < 0.001).

### 4.3. The Differences in the Relationship Between GenAI and Student Engagement Across Learning Contexts

**Figure 3:**
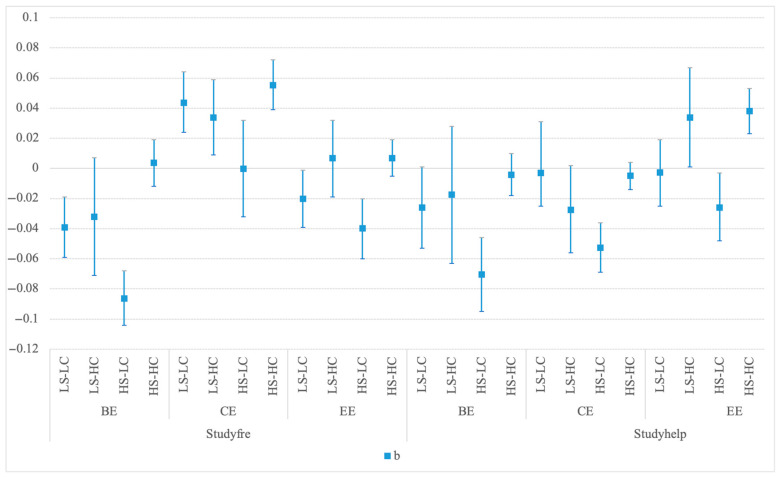
The associations between GenAI use and student engagement in different learning contexts. Notes: The square dots in the figure represent estimated coefficients and the bars represent the related 95% confidence intervals in Model 4. All models include constant and covariates (Female, Ethnic minority, Urban, SES, GKScore, DWC, Major, Grade, SD, and Missing dummies). Standard errors are clustered by higher education institution. LS-LC refers to the “low-support, low-challenge” learning context, LS-HC refers to the “low-support, high-challenge” learning context, HS-LC refers to the “high-support, low-challenge” learning context, and HS-HC refers to the “high-support, high-challenge” learning context.

Figure 3 illustrates the estimated coefficients and related 95% confidence intervals of the relationship between the frequency and satisfaction of GenAI and student engagement across learning contexts. Controlling for other independent variables, the relationships between GenAI and learning engagement across all dimensions vary in different learning contexts.

Specifically, in “low-support, low-challenge” context, the frequency of GenAI use is negatively correlated with behavioral engagement (BE) and emotional engagement (EE), while positively correlated with cognitive engagement (CE). Satisfaction with GenAI’s usefulness is negatively correlated with all three dimensions, though not significantly.

In “low-support, high-challenge” context, the frequency of GenAI use is negatively correlated with behavioral engagement (BE), while positively correlated with cognitive engagement (CE) and emotional engagement (EE). Satisfaction with GenAI’s usefulness is negatively correlated with behavioral engagement (BE) and cognitive engagement (CE), and positively correlated with emotional engagement (EE).

In “high-support, low-challenge” context, both the frequency of GenAI use and the satisfaction with its usefulness are negatively correlated (or not significantly correlated) with all three dimensions.

In “high-support, high-challenge”, the frequency of GenAI use is positively correlated with cognitive engagement, and not significantly correlated with the other two dimensions. Satisfaction with GenAI’s usefulness is positively correlated with emotional engagement (EE), and not significantly correlated with the other two dimensions.

**Figure 4:**
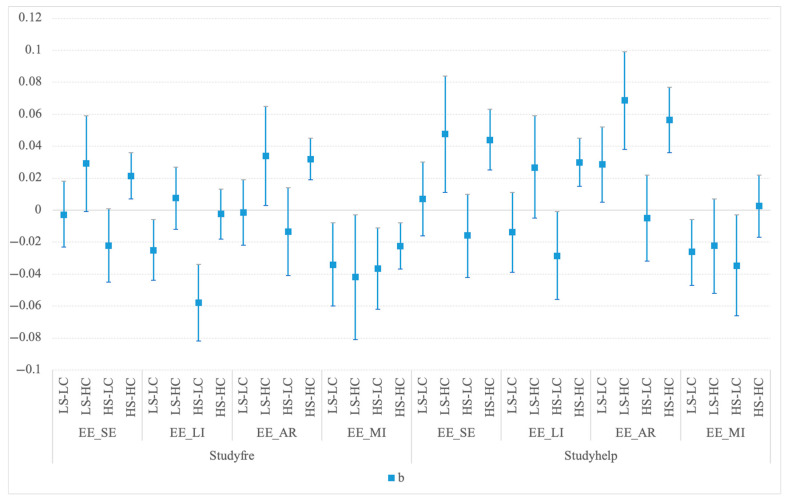
The associations between GenAI use and sub-dimensions of emotional engagement in different learning contexts. Notes: The square dots in the figure represent estimated coefficients and the bars represent the related 95% confidence intervals in Model 4. All models include constant and covariates (Female, Ethnic minority, Urban, SES, GKScore, DWC, Major, Grade, SD, and Missing dummies). Standard errors are clustered by higher education institution. LS-LC refers to the “low-support, low-challenge” learning context, LS-HC refers to the “low-support, high-challenge” learning context, HS-LC refers to the “high-support, low-challenge” learning context, and HS-HC refers to the “high-support, high-challenge” learning context.

Figure 4 illustrates the estimated associations between the frequency and satisfaction of GenAI and the sub-dimensions of emotional engagement across learning contexts. As the figure shows, across all four types of learning contexts, GenAI use is negatively correlated with the level of learning motivation (EE_MI). In high-challenge environments, GenAI use shows a positive association (though sometimes not significant) with self-efficacy (EE_SE), learning interest (EE_LI), and academic resilience (EE_AR). The positive relationship becomes more pronounced when support is low in most cases. In low-challenge learning contexts, GenAI use shows a negative or no significant association with both self-efficacy (EE_SE) and learning interest (EE_LI); however, in “low-support, low-challenge” environments, the satisfaction with GenAI’s usefulness is positively correlated with academic resilience (EE_AR).

## 5. Discussion and Conclusions

### 5.1. Summary of Findings

This study provides one of the first empirical analyses of the use of GenAI and its effect among college students in China, drawing upon large-scale survey data to capture nuanced patterns of use and impact across diverse educational contexts. By leveraging a representative sample covering a wide range of institutions and student backgrounds, the study offers a detailed and timely account of how GenAI is currently being integrated into student learning practices.

First, we found that over sixty percent of participants reported utilizing GenAI technologies, with academic use exceeding daily use. College students most frequently employ GenAI for information retrieval and organization tasks, such as literature searches, reading, and synthesis. However, only approximately one-fifth of users believe that GenAI can fulfill 80% of their needs when engaging in such academic tasks, a figure notably lower than its perceived usefulness for managing everyday life. This pattern of high use but modest perceived academic utility suggests that while GenAI has become a visible presence in students’ academic routines, its reliability and depth of function remain limited, possibly due to issues related to output accuracy and ethical uncertainties in the academic context (Essien et al., 2024). These findings provide an evidence-based foundation for international comparisons, contributing empirical granularity to broader debates about the pedagogical potential and constraints of GenAI.

Second, this study further examines the association between GenAI use and student learning engagement. Table 4 below summarizes the directions of the estimated associations. According to the first panel, students who have used GenAI show a higher level of cognitive and emotional engagement (especially in academic self-efficacy and resilience) than those who have not. However, further findings demonstrate that the relationships between GenAI use and student engagement are far more complex.

The results shown in Panel 2 only partly support our Hypotheses 2 and 3, and deny the Hypothesis 1. These findings indicate that GenAI use has replaced activities such as previewing, note-taking, and reviewing in traditional learning. The frequency of GenAI use in academic activities and engagement in higher-order thinking is mutually reinforcing. Students who perceive GenAI as helpful for learning exhibit more positive emotional engagement; however, the frequency of GenAI in academic settings also implies lowered interests and motivation in learning.

Considering different learning contexts further complicates the relationships. As shown in Panel 3, the role of GenAI varies by learning contexts. Overall, it facilitates learning in high-challenge contexts and leads to “perfunctory” learning in low-challenge ones, yet it replaces behavioral engagement to some extent in both cases. GenAI use is most positively oriented in the “high-support, high-challenge” context and most negatively oriented in the “high-support, low-challenge” environment. The findings overall support Hypothesis 4.

### 5.2. Discussion and Implications

The above findings reveal how Chinese college student utilize GenAI in their study. In most cases, students use GenAI in a way to replace routine work and facilitate higher-order learning in a high-challenge environment. This supports prior assertions that technology, when effectively integrated, can serve as a cognitive amplifier (Chiu, 2023), enabling learners to engage more deeply with content and sustain effort under challenging conditions. However, the study also finds a potential risk of over-reliance on the GenAI, which could lead to diminished learning interest and motivation. This asymmetry between perceived utility and actual behavioral outcomes reflects recent critiques of “technological solutionism” in EdTech discourse (Selwyn, 2016). In sum, these findings suggest that the current capacity of GenAI to empower student learning remains relatively limited, highlighting the necessity for enhanced design improvements to better align with and meet the specific learning needs of students.

Moreover, the role of GenAI is shown to be highly context-dependent, varying with the characteristics of learning environments. These results affirm and expand upon existing theoretical models of educational technology adoption (Keuning et al., 2024), highlighting that the utility of GenAI is not intrinsic but contingent on how well its affordances fit with pedagogical design. In this light, we propose a conditional effectiveness model of GenAI in higher education, wherein its positive impact is maximized when deployed as a complementary cognitive resource embedded within high-quality human teacher instruction. Contrary to concerns about AI displacing educators (Belda-Medina & Kokošková, 2023; Wang et al., 2023; Yan et al., 2023), our findings underscore the enduring and indeed amplifying role of educators in fostering meaningful student engagement.

Taken together, these findings carry several important implications for educational policy and instructional practice. First, they call for a shift away from one-size-fits-all approaches to GenAI governance in universities. Rather than blanket prohibitions or unconditional endorsement, institutions should develop context-sensitive strategies that align GenAI integration with the pedagogical intent and structural features of specific courses. Second, these results reaffirm the centrality of instructors in orchestrating effective learning environments, particularly in the age of AI, and highlight the need for faculty development initiatives that enable educators to meaningfully incorporate GenAI into their teaching practices. As GenAI continues to evolve, equipping students not only with access but with the meta-cognitive skills necessary to manage their engagement with AI responsibly and productively becomes an essential task for higher education.

It is important to acknowledge the limitations of this study. The sample contains more elite universities, potentially limiting the generalizability of our findings to the broader student population. The over-representation may lead to overestimation of both the prevalence and impact of GenAI use, as students in elite universities might be more engaged with emerging technologies compared with their peers at less-resourced institutions. In addition, the self-selection of GenAI users may introduce endogeneity, as students who choose to use GenAI may differ systematically in their motivation, aptitude, or digital literacy. Therefore, the findings of the current study could not be interpreted as causal relationships. Future studies should adopt experimental or quasi-experimental designs and longitudinal data to more robustly identify causal effects over time, and expand sampling to encompass a broader spectrum of institutions. Furthermore, the study is limited to China. While we believe the core findings may have some universal relevance, the absence of international comparative data limits our ability to contextualize our findings within the global landscape.

Despite these limitations, the present study advances theoretical and empirical understanding of GenAI in higher education by showing that its effectiveness is fundamentally conditioned by the instructional design of human teachers. GenAI, in its current state, should be viewed not as a pedagogical or learning replacement, but as a contingent supplement (Rahman & Watanobe, 2023)—its value residing not in its standalone capacity, but in how well it is integrated within human-centered educational systems.

## Figures and Tables

**Table 1:** Demographic characteristics of the sample.

Variable	Definition and Measurement	M(SD)/%	Missing Rate (%)
Female	Gender. Binary: 1 = Female,	47.80%	0
	0 = Male.	52.20%	
Ethnic minority	Ethnicity. Binary: 1 = Ethnic Minority,	8.51%	0
	0 = Han.	91.49%	
Urban	Living region. Binary: 1 = Urban,	73.36%	0
	0 = Rural.	26.54%	
SES	Socio-Economic Status of family. Constructed with self-reported family income, educational level and career of parents by factor analysis. Continuous.	−0.03(0.98)	1.23
GKScore	Standardized GaoKao score by province, exam year, and examination type. Continuous.	0.06(0.96)	2.37
DWC	Study in Double World-Class (DWC) HEIs. Binary: 1 = yes, 0 = no.	61.13%	0
		39.87%	
Grade	Year in college Categorical: 1–4 = year 1–4.	31.84%	0
		27.13%	
		23.84%	
		17.20%	
Major area	Area of an academic major in college. Categorical: 1 = Humanities,	9.23%	0
	2 = Social sciences,	34.51%	
	3 = Sciences,	10.24%	
	4 = Engineering (not computer science),	35.51%	
	5 = Engineering (computer science).	10.52%	

**Table 2:** Academic use of GenAI in learning contexts.

Variable	M (SD) of z-Score				ANOVA Test F-Value	Bonferroni Post-Hoc Test
	1 Low-Support and Low-Challenge	2 Low-Support and High-Challenge	3 High-Support and Low-Challenge	4 High-Support and High-Challenge		
Studyfre	−0.12 (0.86)	0.10 (1.02)	−0.15 (0.92)	0.13 (1.11)	251.89 ***	4 = 2 > 1 = 3
Studyhelp	−0.17 (0.89)	0.02 (0.98)	−0.07 (0.96)	0.17 (1.08)	380.34 ***	4 > 2 > 3 > 1

Note: *** *p* < 0.001; = indicates no significant difference was found between the groups at the 0.01 level; > indicates the mean score of the former group was statistically significantly larger than that of the latter at the 0.01 level.

**Table 3:** The estimated association between GenAI use and student engagement.

	BE	CE	EE	EE_SE	EE_LI	EE_AR	EE_MI
	b/se	b/se	b/se	b/se	b/se	b/se	b/se
Panel 1. The overall use of GenAI							
AIuse	−0.0073	0.0559 ***	0.0266 **	0.0287 **	0.0205	0.0345 **	0.0080
	(0.0086)	(0.0082)	(0.0083)	(0.0087)	(0.0099)	(0.0101)	(0.0093)
N	72,615	72,615	72,615	72,615	72,615	72,615	72,615
R-squared	0.3506	0.3949	0.3084	0.2595	0.2512	0.2522	0.1442
Panel 2. The frequency and satisfaction of GenAI in different settings							
Studyfre	−0.0214 **	0.0444 ***	−0.0061	0.0100	−0.0143 *	0.0171 **	−0.0291 ***
	(0.0058)	(0.0064)	(0.0039)	(0.0055)	(0.0054)	(0.0045)	(0.0062)
Studyhelp	−0.0194 **	−0.0090	0.0174 *	0.0255 **	0.0088	0.0416***	−0.0128
	(0.0064)	(0.0064)	(0.0080)	(0.0089)	(0.0078)	(0.0092)	(0.0076)
Lifefre	0.0889 ***	0.0381 ***	0.0720 ***	0.0587 ***	0.0967 ***	0.0257**	0.0572 ***
	(0.0070)	(0.0059)	(0.0080)	(0.0085)	(0.0076)	(0.0083)	(0.0075)
Lifehelp	0.0289 ***	0.0252 ***	0.0645 ***	0.0643 ***	0.0650 ***	0.0520 ***	0.0367 ***
	(0.0071)	(0.0044)	(0.0073)	(0.0074)	(0.0069)	(0.0082)	(0.0072)
N	45,368	45,368	45,368	45,368	45,368	45,368	45,368
R-squared	0.3582	0.3990	0.3236	0.2756	0.2725	0.2601	0.1462

Note: All models include constant and covariates (Learning contexts (three dummy variables), Female, Ethnic minority, Urban, SES, GKScore, DWC, Major, Grade, SD, and Missing dummies). Robust standard errors, clustered by higher education institution, are reported in parentheses. * *p* < 0.05, ** *p* < 0.01, *** *p* < 0.001.

**Table 4:** Summary of the findings.

	BE	CE	EE	EE_SE	EE_LI	EE_AR	EE_MI
1. Ever used GenAI	0	+	+	+	0	+	0
2. GenAI use in academic settings	−/−	+/0	0/+	0/+	−/0	+/+	−/0
3. In different learning contexts							
Low-support and low-challenge	−/0	+/0	−/0	0/0	−/0	0/+	−/−
Low-support and high-challenge	0/0	+/0	0/+	0/+	0/0	+/+	−/0
High-support and low-challenge	−/−	0/−	−/−	0/0	−/−	0/0	−/−
High-support and high-challenge	0/0	+/0	0/+	+/+	0/+	+/+	−/0

Note: “0” indicates non-significance, “+” represents positive significance, and “−” denotes negative significance. Before the “/”, the coefficient represents the impact of the frequency of AI use in academic tasks (Studyfre), and after the “/”, it represents the coefficient of the perceived usefulness (Studyhelp).

## Data Availability

The data presented in this study are available upon request from the corresponding author.

## Notes

1 The Double World-Class Initiative, initiated in 2015, is a higher education development and sponsorship scheme of the Chinese central government. It is a highly selective elite university project recruiting only 147 out of 2820 regular HEIs by 2024, all of which are public-funded.

2 We used Confirmatory Factor Analysis (CFA) to examine the structural validity of the indicators. All indicators performed well. The results were reported in the CCSS 2024 Handbook and are available upon request.

## Appendix A

**Table A1:** Definition, measurement, and descriptive statistics of variables (N = 72,615).

Variable	Definition and Measurement	M(SD)/%	Missing Rate (%)
Outcomes			
BE	Behavioral engagement. Constructed with self-reported frequency of taking active learning behaviors, including concentrating on teacher’s lecture in class, taking notes in class, active participation in class discussion, on-time review and summarizing, etc. (6 items, Cronbach’s alpha = 0.91).	67.23(22.01)	0
	Continuous, scale: 1–100		
CE	Cognitive engagement. Constructed with self-reported frequency of taking integrative thinking, reflective thinking, and critical thinking behaviors in learning (9 items, Cronbach’s alpha = 0.95).	68.22(21.16)	0
	Continuous, scale: 1–100		
EE	Emotional engagement. Constructed with the arithmetic average of self-reported scores on EE_SE, EE_LI, EE_AR and EE_MII.	69.63(17.56)	0
	Continuous		
EE_SE	Students’ beliefs in their own ability to gain academic achievements (3 items, Cronbach’s alpha = 0.85).	69.51(19.93)	0
	Continuous, scale: 1–100		
EE_LI	Learning interest (2 items, Cronbach’s alpha = 0.77).	67.11(21.77)	0
	Continuous, scale: 1–100		
EE_AR	The psychological resilience when faced with academic challenge and pressure (4 items, Cronbach’s alpha = 0.89).	70.98(19.46)	0
	Continuous, scale: 1–100		
EE_MI	The intensity of learning motivation measured by a 7-point Likert-scale item and transformed into a 100-point variable.	70.90(22.43)	0
	Continuous, scale: 1–100		
Key explanatory variables			
AIuse	Whether has used GenAI	64.47%	0
	Binary: 1 = Yes, 0 = No.		
Studyfre	Academic utilization frequency of GenAI. Constructed with the arithmetic average of utilization frequency of GenAI addressing with academic tasks, including computational tasks (e.g., coding, solving mathematical problems, analyzing data), information retrieval and synthesis (e.g., conducting literature reviews, organizing data), and writing or creative tasks (e.g., generating paper outlines, developing research proposals). (3 items, Cronbach’s alpha = 0.86).	49.36(24.02)	35.60
	Continuous, scale: 1–100		
Studyhelp	Academic help from GenAI. Constructed with the arithmetic average of perceived usefulness of GenAI addressing academic tasks (3 items, Cronbach’s alpha = 0.87).	59.98(24.36)	36.84
	Continuous, scale: 1–100		
Lifefre	Utilization frequency of GenAI in daily life. Constructed with the arithmetic average of utilization frequency of GenAI addressing with daily tasks, including managing everyday activities (e.g., searching for general information, planning travel itineraries) and handling administrative duties (e.g., completing forms, drafting standardized documents). (2 items, Cronbach’s alpha = 0.70).	43.55(26.41)	35.55
	Continuous, scale: 1–100		
Lifehelp	Help from GenAI in daily life. Constructed with the arithmetic average of perceived usefulness of GenAI addressing daily tasks (2 items, Cronbach’s alpha = 0.72).	56.14(27.21)	37.29
	Continuous, scale: 1–100		
Course type	The type of courses, measured by the level of support and challenge. Categorical: 1 = courses with low support and low challenge 2 = courses with low support and high challenge 3 = courses with high support and low challenge 4 = courses with high support and high challenge	38.96%	0
		6.75%	
		11.22%	
		43.06%	
Covariates			
SD	Social desirability	56.76(28.51)	0
	Continuous scale: 1–100		
All variables shown in Table 1. (Repetition is omitted here.)			
